# Loss of balancing selection in the βS globin locus

**DOI:** 10.1186/1471-2350-11-21

**Published:** 2010-02-03

**Authors:** Niven A Salih, Ayman A Hussain, Ibrahim A Almugtaba, Abeir M Elzein, Ibrahim M Elhassan, Eltahir AG Khalil, Hani B Ishag, Hiba S Mohammed, Dominic Kwiatkowski, Muntaser E Ibrahim

**Affiliations:** 1 Department of Molecular Biology, Institute of Endemic Diseases, Medical Campus, Qasser Street, University of Khartoum, Khartoum, Sudan; 2 School of Mathematical Sciences, University of Khartoum, Khartoum, Sudan; 3 Wellcome Trust Centre for Human Genetics, Roosevelt Drive Headington, Oxford, OX3 University of Oxford, UK

## Abstract

**Background:**

Probably the best example of the rise and maintenance of balancing selection as an evolutionary trend is the role of S-haemoglobin (HbS - rs334) in protecting from malaria. Yet, the dynamics of such a process remains poorly understood, particularly in relation to different malaria transmission rates and the genetic background of the affected populations.

**Methods:**

We investigated the association of haemoglobin HbS in protection from clinical episodes of malaria in two populations/villages where malaria is endemic, but mostly presenting in mild clinical forms. Five-hundred and forty-six individuals comprising 65 and 82 families from the Hausa and Massalit villages respectively were genotyped for HbS. Allele and genotype frequencies as well as departure from Hardy-Weinberg Equilibrium were estimated from four-hundred and seventy independent genotypes across different age groups. Age-group frequencies were used to calculate the coefficient-of-fitness and to simulate the expected frequencies in future generations.

**Results:**

Genotype frequencies were within Hardy-Weinberg expectations in Hausa and Massalit in the total sample set but not within the different age groups. There was a trend for a decrease of the HbS allele frequency in Hausa and an increase of frequency in Massalit. Although the HbS allele was able to confer significant protection from the clinical episodes of malaria in the two populations, as suggested by the odds ratios, the overall relative fitness of the HbS allele seems to have declined in Hausa.

**Conclusions:**

Such loss of balancing selection could be due to a combined effect of preponderance of non-clinical malaria in Hausa, and the deleterious effect of the homozygous HbS under circumstances of endogamy.

## Background

Polymorphisms in the β-globin gene leading to sickle-cell anaemia and thalassemia are among the most notable examples of balancing selection in the human genome. Over half a century ago Haldane [1], suggested that protection against malaria was conferred by an abnormal form of haemoglobin known as thalassemia. It was later suggested that these deleterious mutations are maintained in the population in a state of balanced polymorphism because of the protective effect against severe forms of malaria conferred by the heterozygous states [2-4]. The observed overlaps in the geographic distributions of malaria with the haemoglobinopathies, particularly HbS [5-7], have been cited in support of the hypothesis that malaria has been an important evolutionary force in selection of this and other variants, with evidence for genome-shaping interactions found in the geographic and ethnic distributions across the globe [8]. However, it is not clear what happens to these polymorphisms in areas where the selective pressure has become relaxed or is nonexistent altogether. In some cases, as in the Caribbean, the HbS mutation has been shown to continue to exist with unaltered frequency [9], despite the near eradication of malaria more than half a century ago.

Here we study the behaviour of the HbS polymorphism in two village populations of different ethnic origins, in an attempt to understand the evolutionary trajectory of this mutation under weak natural selection, where malaria is endemic but has a mostly mild clinical presentation.

## Methods

### Area and populations

Um-Salala village is located on the eastern bank of the River Rahad about 400 kilometres south-east of Khartoum. It is inhabited by the Massalit tribe who migrated from El-Geneina in Darfur state, Western Sudan, and settled along the Rahad River. The migration took place mostly during the 1980s when drought and famine struck Darfur. The Massalit language is part of the Nilo-Saharan family of languages.

Koka village was established 50 years ago by Hausa, an Afro-Asiatic Speaking ethnic group originally from northern Nigeria, and is located around 40 kilometers east of Um-Salala. The area is endemic for both malaria and visceral leishmaniasis (VL). The Koka community is a relatively closed community with above 90% of all marriages in the past 40 years occurring within the village versus 28% of marriages among the Massalit being within village. Polygamy is common in both villages but consanguinity is rather occasional.

### Ethical considerations

The study was reviewed and ethically approved by the Ethical Committee of the Institute of Endemic Diseases, University of Khartoum; Samples were collected following written informed consent from all individuals or their guardians.

### Study design

Eight surveys were carried out in the two villages from 1994 to 2006. During these surveys the inhabitants of the two villages were interviewed and the clinical and parasitological data collected on pre-designed forms. In 2004 an active surveillance system was introduced in the two villages for monitoring clinical malaria.

Classification of malaria cases was based on the symptoms, physical signs, and laboratory findings of malaria. Uncomplicated clinical malaria was defined as any parasitaemia with fever (body temperature more than 37°C or reported fever). Severe malaria was defined according to WHO criteria as malaria that presents with life-threatening conditions including coma, severe anemia, hypoglycemia, shock or convulsions. Asymptomatic malaria was defined as a positive blood film or Immuno-Chromatography Test for malaria parasite in the absence of symptoms.

### Genotyping

DNA was extracted from buccal samples using the chloroform method and the HbS polymorphism (rs334) was assayed using allele-specific PCR primers as previously described [10]. Briefly, separate PCR reactions were set up for the HbA and HbS alleles and each reaction contained an additional pair of primers, designed to amplify conserved region of the genome, to act as an internal control. The PCR products were detected following 2% agarose gel electrophoresis. Genotypes were confirmed using Cellulose-Acetate haemoglobin electrophoresis (Alkaline pH 8.5) with Ponceau Red staining, yielding clear discrimination of haemoglobin A, F, S, C and A_2_.

### Statistical analysis and simulation

Allele and genotypes frequencies of HbA and HbS were calculated and compared using Chi Square Test and F statistics. Departure from Hardy-Weinberg Expectations (DHWE) was calculated using Chi Square Test. Odds ratios of the protective effect of carrying the sickle cell allele on clinical malaria cases versus asymptomatic cases were calculated in the two populations. The Odd Ratios of carrying also the alleles of hemoglobin S on clinical cases versus carrying the HbS allele in mild or no malaria group were calculated using the VASSARSTAT, exact 95% confidence limit (CL). The p value, *P *< .05 was considered statistically significant. The expected alleles and genotype frequencies of HbA, HbS were simulated for fifty generations using **MATLAB **student version 5.3.

The allele Fitness and the change of the frequencies in the next generation were calculated using the following formula:

Where p' is the is the frequency of the A allele in the second (offspring) generation, p is its frequency in the first (parental) generation and q is the frequency in the first generation of the S allele. The relative fitness of the heterozygote is taken to be (1+h), and the relative fitness of the AA homozygote to be 1 and that of the SS homozygote to be 0.

## Results

**Figure 1:**
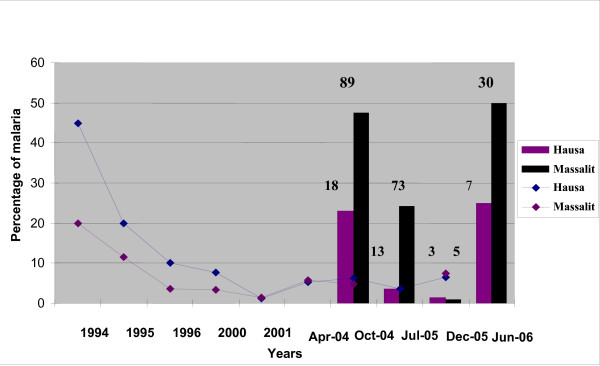
**Prevalence of malaria in the two villages, 1994-2006**. Lines define the prevalence during eight surveys from 1994 to 2005. Bars show the proportion of clinical malaria cases to the total febrile episodes as percentage in the two populations during follow up intervals in 2004, 2005 and 2006. Numbers on top of the bars indicate the actual number of cases of clinical malaria.

Prevalence of Malaria in the two villages during eight cross sectional surveys between 1994 and 2005 is shown in figure 1. All cross sectional surveys prior to 2004 showed little clinical malaria in the two villages. From 2004 onwards, the cross sectional surveys were supplemented by active surveillance involving village workers and teams that stayed over during the rainy season. Cases of malaria with clinical symptoms (mostly non severe) showed up more profoundly in the Massalit. Clinical malaria was consistently few folds higher in Massalit than Hausa (Figure 1).

Distribution of malaria in different age groups in Hausa and Massalit is shown in Table 1. The highest numbers of cases were found in the (0-15) age group, although in the Massalit village, there was more malaria in adults compared to the Hausa village.

**Table 1:** Age distribution of Hb genotypes and HWE of Hausa and Massalit populations (Percentages from Total bracketed):

	Genotypes							
	Hausa			DHWE	Massalit			DHWE
**Age groups**	**AA**	**AS**	**SS**		**AA**	**AS**	**SS**	
**N**	**118**	**91**	**15**		**163**	**68**	**15**	
								
0_15	35(54)	23(35)	7(11)	F = 0.13 X^2 ^= 1.12 P = 0.3	42(48)	34(39)	11(13)	F = 0.10 X^2 ^= 0.95 P = 0. 33
								
16_30	65(57	41(36)	8(7)	F = 0.04 X^2 ^= 0.19 P = 0.66	40(68)	15(25)	4(7)	F = 0.19 X^2 ^= 2.13 P = 0.14
								
> 30	18(40)	27(60)	0(0)	F = -0.4 X^2 ^= 8.2 P = 0.004	81(81)	19(19)	0(0)	F = -0.10 X^2 ^= 1.10 P = 0.29

Clinical malaria was mainly manifested as parasitemia with a body fever of 38-42°C, headaches and occasionally with vomiting and diarrhoea. Consistent with low clinical malaria burden, anaemia was found to be infrequent in the two populations; the mean haemoglobin level in the Hausa population was 14.2 ± 1.08 g/dl and for Massalit 13.1 ± 1.78. In Figure 1, clinical malaria during follow up is depicted both as a percentage of the total febrile cases reported and as total numbers and was consistently higher in Massalit than Hausa.

A total of 546 individuals (261 from Hausa and 285 from Massalit) were screened for the normal haemoglobin HbA and haemoglobin HbS variants. The frequencies of the two alleles were calculated for a sample of 470 individuals who were not direct first degree relatives to ensure genotype independence (224 Hausa and 246 Massalit). The frequencies of haemoglobin A and S, for all individuals, were 0.76 and 0.25 in Hausa and 0.80 and 0.20 in Massalit respectively. Genotype frequencies were as follows: 0.53, 0.41, and 0.6 for AA, AS, and SS respectively in Hausa and 0.66, 0.28, 0.6 for Massalit. Genotypes were in Hardy-Weinberg Equilibrium for both Hausa and Massalit population (*P = 0.91 *and *P = 0.11 *respectively). Frequencies of HbA and HbS genotypes were calculated in three different age groups (Table 1), although those over and below 30 years were considered in a conservative estimate for splitting the population into two successive generations for the analysis in calculating the fitness values. The frequencies of HbA and HbS in the first generation in Hausa were respectively 0.67, 0.22 and 0.12, and for the next generation the frequencies were 0.66, 0.27 and 0.099. In Massalit the frequencies in the first generation were 0.77, 0.13 and 0.11 respectively while the frequencies in the next generation were 0.60, 0.29 and 0.11 (Table 1). Genotypes were within HWE for both Hausa and Masalit for the < 30 age group. However in the >30 age group there was DHWE both by X^2 ^(*P = 0.004*) and a negative F statistics (-0.4) in Hausa and a negative F value (-0.10) in Massalit.

The frequency of HbS in the two groups of clinical and asymptomatic malaria was studied to determine its protective effect (Table 2). The odd ratios in Hausa for the protective effect of HbS in clinical malaria versus population control (no malaria group was: OR = 3.98; *P *= 0.01, while the odd ratio for asymptomatic malaria versus clinical malaria was: OR = 8.18; *P *= < 0.0001. In Massalit the odd ratio for the protective effect of HbS in clinical malaria versus asymptomatic was: OR = 3.53, *P *= 0.001, while the odd ratio for clinical malaria versus population controls was: OR: 2.48, *P *= 0.004.

**Table 2:** Calculated odds ratios from the prevalence of clinical and asymptomatic malaria in AS, SS individuals in Massalit and Hausa:

	HbA	HbS	HbS Allele Frequency	Odds Ratio (95%Cl)	Relative Risk (95% Cl)	*P*.value
**Hausa**						
All malaria	76	57	0.4286			
Clinical malaria	46	9	0.1636			
Asymptomatic malaria	30	48	0.6515	8.178(3.504- 19.088)	2.133(1.58- 2.88)	< 0.0001
Population control	72	56	0.4357	3.975(1.795- 8.801)	1.412(1.186- 1.68)	0.0003
						
**Massalit**						
All malaria	96	37	0.2782			
Clinical malaria	70	16	0.1860			
Asymptomatic malaria	26	21	0.4468	3.534 (1.602- 7.794)	2.096(1.360- 3.229)	0.001
Population control	97	55	0.3618	2.481(1.313- 4.686)	1.334(1.114- 1.597)	0.004

**Figure 2:**
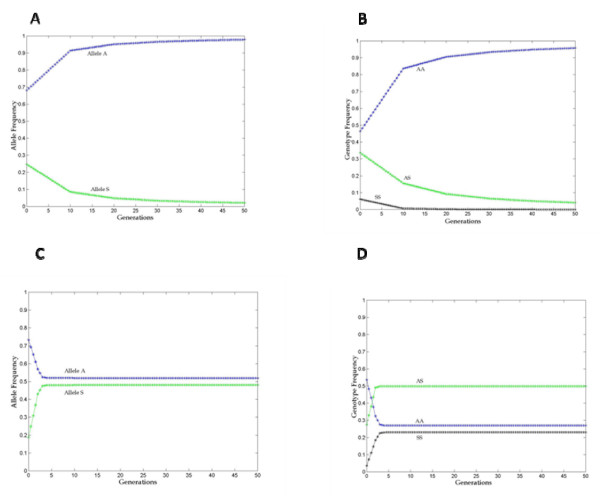
**Simulation of allele and genotype frequencies of the βS polymorphism for 50 generations in Hausa and Massalit villages**. Simulation is based on actual genotype data and with projections based on fitness equation as described in the materials and methods section. Figure 2(A): Simulation for HbA and Hb S allele frequencies over 50 generations in Hausa. Figure 2(c): Simulation for HbA and Hb S alleles frequencies over 50 generations in Massalit. Figure 2(B): Simulation for HbA and Hb S genotypes frequencies over 50 generations in Hausa. Figure 2(D): Simulation for HbA and Hb S genotypes frequencies over 50 generations in Massalit. All simulations are based on fitness measures as calculated from genotypes of two generations.

Based on the allele frequencies of those over 30 years old, considered as the parental generation and those 30 years or less taken as their progeny (P'), simulations using the programme **Matlab**, were carried out to project allele (Figures 2A and 2C) and genotype (Figures 2B and 2D) frequencies in future generations. The results show the allele frequencies of HbA increasing to 0.92 and HbS decreasing to 0.08 in Hausa within 10 generations. The simulated frequencies of HbA and HbS alleles for Massalit, reached equilibrium (0.52 for HbA and0.48 for HbS) in the fourth generation(Figure 2). Both simulations assume a simplified model of steady transmission of malaria and a constant population size for the Massalit and Hausa. The genotype simulations predict persistence of rs334 in both AS and SS forms at low frequency in Hausa until the 50^th ^generation even with this current population parameters and fitness values. In the Masalit, however, the population is projected to reach equilibrium in less than 5 generations.

## Discussion

The two populations of Hausa and Massalit moved to the area of the Rahad River within the past 50 years. The area used to be a game reserve where both VL and malaria were endemic as well as other infectious and water borne diseases, making the settlement a challenging and costly undertaking in terms of health. The Massalit in particular succumbed to VL in what appears to be a case of extreme natural selection, as evidenced by mortality records in adults and children.

The area is endemic for malaria with mostly unstable transmission. Longitudinal follow up of these two populations has indicated that despite malaria endemicity, the disease generally presents in mild clinical forms particularly in Hausa. The recent West African origin and agricultural life style of the Hausa imply an early and stable exposure to the malaria parasite with extended periods of co-evolution.

In our surveys the overall prevalence of malaria (mostly asymptomatic) was in decline since 1994, possibly due to dynamics of immunity to malaria infection in the population. In Massalit the trend appeared similar to Hausa, but the incidence of clinical malaria was consistently higher both in numbers and as a proportion of the total febrile cases. Anaemia is virtually nonexistent in both populations with average haemoglobin of 14.2 g/dl in Hausa and 13.1 g/dl in Massalit, except for the few homozygous HbS cases. The HbS or sickle-cell mutation was the primary candidate to explore to explain the protection seen against the clinical manifestations of *Plasmodium falciparum *malaria. The allele frequency of the S allele was found to be high in both populations (0.25 in Hausa and 0.20 for Massalit) which was rather surprising for the Massalit, a group indigenous to Darfur of western Sudan where we [10,11] and others [12], have indicated based on analysis of the HbS haplotypes and other markers, a recent introduction of HbS into Sudan and the Eastern Sahel. This is also consistent with the hypothesis of a recent origin of *P. falciparum *as a major human pathogen emerging with the beginnings of agriculture, when human populations started to form resident communities that allowed the establishment of substantial reservoir of infection [8,13,14].

Although the allele frequency were in overall comparable between populations, the distribution of genotypes and alleles across age groups, was different between Hausa and Massalit, with a trend of increase in allele frequency in Massalit over generations and a decrease in Hausa. There was departure from HWE in both Hausa and Massalit in the >30 age group consistent with an effect of balancing selection over time. DHWE could be explained both from the perspective of natural selection or population effects, which are often interacting and difficult to disentangle. It has been proposed that sub-structured populations would have different (N_e_) values and would behave in a panmictic manner [15]. The closed nature of the Hausa villages where within village marriages was >90% versus ~30% in Massalit might have equally influenced the alleles fate. The impact of endogamy on the segregation of HbS and similar traits needs to be carefully examined given the existing contrasting views on its impact on these variants. In one study it has been shown that under selection pressure from malaria, consanguinity and endogamy may have increased the speed of selection of alpha thalassemia [16], while another study reported no obvious role for consanguinity in the distribution of genetic traits related to malaria [17].

We also note here the remarkably high frequency of the AS genotype in the below 1-15 age group in the Massalit (39% of the total age group and 50% of the total heterozygosity). Considering the heavy death toll of VL outbreaks on Massalit children in the past years, one cannot rule out a possible added selective advantage for HbS against VL which could also be viewed in the recent reports of abnormal haemoglobin influencing the production of nitric oxide, a main effecter molecule in the patho-physiology of both malaria and leishmaniasis [18], or a scenario analogous to that of thalassemia protecting against bacterial and other infections [19].

A simulation of allele frequencies over 50 generations have predicted haemoglobin S to diminish to a virtual complete loss of the allele by the 30th generation in Hausa, whereas in Massalit, the simulation predicts that the alleles will reach equilibrium by the fourth generation and be maintained while the selective pressure exits. The nature of this selective pressure is still poorly understood. The fitness of the different genotypes and their fate in models proposed so far [1-3] is a direct function of mortality caused by the malaria parasite, although indirectly inferred from the general mortality indices.

The protective advantage conferred by the HbS gene against falciparum malaria shown by values of odds ratio in this study, is comparable in Hausa and Massalit, although the overall incidence of clinical malaria is considerably higher in Massalit as seen by active surveillance over the past three years. The opposite seems to be the case in Hausa where non-clinical manifestation of falciparum malaria is commoner. Although the odds ratio and distribution of clinical versus asymptomatic malaria, shows the protective role of the HbS allele among those suffering from clinical diseases, the fact that clinical malaria does occur in a disproportionately lower portion of the population, might have given the selective advantage of the sickle cell trait an inferior advantage in the total population.

The presence of hitherto unknown protective polymorphism(s) that may provide a slightly elevated fitness, as is the case with HbC [20], cannot be ruled out. In this case, and as stated in the malaria hypothesis, the HbS mutation will be at a disadvantage with a continuing deleterious impact in the homozygous SS state. Interestingly, no substantial decline in the frequency of the HbS gene was ever reported before, even in areas where malaria selective pressure is no longer in operation [9]. This might be due to the large population size and the panmictic nature of the populations in question, where random mating and a relatively large population size might have helped extend the frequency of the HbS allele in time.

## Conclusion

We provide evidence of a significant change in allele frequency in the HbS locus that could be observed within 2-3 generation in the presence of malaria transmission. In the Hausa village, this seems to be likely due to the low clinical burden of the disease, the population effect (possibly under drift) in addition to the deleterious impact of the homozygous HbS, both conspiring against the maintenance of balancing selection. In the Massalit village, the relatively higher episodes of clinical malaria, in addition to a potential impact from visceral leishmaniasis (a disease with a higher fatality rate), may be responsible for the different selection profile.

## Competing interests

The authors declare that they have no competing interests.

## Authors' contributions

NS, Collected and analysed the data and contributed to the writing of manuscript; IA, Carried out the simulations; AME carried out the QC genotyping; AH, Analysed the data and contributed to the manuscript writing;, IME contributed to the filed activity; EAGK, patients investigation and care; HI, carried out the protein electrophoresis; HSM contributed to the analysis and laboratory work; DK supervised the QC analysis; MEI conceived of the study supervised the analysis and wrote the manuscript. All authors approved the final manuscript.

## Pre-publication history

The pre-publication history for this paper can be accessed here:

http://www.biomedcentral.com/1471-2350/11/21/prepub
